# Complete chloroplast genomes provide insights into evolution and phylogenetic relationships of *Stachyurus* (Stachyuraceae)

**DOI:** 10.3389/fpls.2026.1734802

**Published:** 2026-05-18

**Authors:** Xinhao Liu, Ruisen Lu, Pan Li, Yingxiong Qiu, Bo Xu, Zhaoping Yang, Yu Feng

**Affiliations:** 1 College of Life Sciences and Technologies, Tarim University, Aral, China; 2 Mountain Ecological Restoration and Biodiversity Conservation Key Laboratory of Sichuan Province, Chengdu Institute of Biology, Chinese Academy of Sciences, Chengdu, China; 3 Institute of Botany, Jiangsu Province and Chinese Academy of Sciences, Nanjing, China; 4 Key Laboratory of Biodiversity and Environment on the Qinghai‐Tibetan Plateau, Ministry of Education, School of Ecology and Environment, Xizang University, Lhasa, China; 5 Wuhan Botanical Garden, Chinese Academy of Sciences, Wuhan, China; 6 State Key Laboratory Incubation Base for Conservation and Utilization of Bio-Resource in Tarim Basin, College of Life Science and Technology, Tarim University, Aral, China

**Keywords:** Chloroplast capture, chloroplast genome, comparative analysis, phylogenetic reconstruction, stachyuraceae

## Abstract

*Stachyurus*, the sole genus of the Stachyuraceae family, comprises approximately 10 species distributed across eastern Asia, with potential applications in horticulture and medicine. However, limited genetic resources have hindered comprehensive research on this genus. Here, we sequenced and assembled the complete chloroplast genomes (cpDNAs) of 25 *Stachyurus* accessions, representing seven currently accepted species and one variety. The assembled *Stachyurus* cpDNAs range from 161,624 to 162,947 bp in length, with a GC content of 37.0–37.1% and 129–131 genes. Comparative analyses revealed a high degree of conservation in genome size, structure, inverted repeat (IR) boundary dynamics, GC content, and repeat patterns. Nevertheless, subtle variations driven by IR boundary shifts, gene loss (particularly *ycf15*), and differences in long sequence repeats collectively account for the observed cpDNA length variation. Seven hypervariable regions as promising molecular markers and four genes exhibited signatures of positive selection were identified. Phylogenetic analyses based on complete cpDNAs showed widespread paraphyly and strong cytonuclear discordance with previous nuclear phylogenies, providing clear evidence of chloroplast capture associated with hybridization. While reciprocal monophyly between Chinese and Japanese species was supported, relationships within the Chinese clade remain poorly resolved. These findings underscore the limitations of plastid data alone and highlight the urgent need for integrative studies combining nuclear genomic data, dense population sampling, and morphological/ecological information to clarify species boundaries and taxonomic relationships within *Stachyurus*. This study provides essential chloroplast genomic resources for future phylogenetic, phylogeographic, and adaptive evolution research on this understudied genus.

## 1 Introduction

*Stachyurus* is the sole genus within the family Stachyuraceae, an ancient shrub family endemic to East Asia and the Himalayan region, ranging from Japan and the Korean Peninsula to southwestern China and the eastern Himalayas (Ohi et al., 2003; Yang and Stevens, 2007; Oh et al., 2021). Southwestern China serves as the core area of species diversity for this genus (Shan, 1999). The latest taxonomy recognizes eight species and three varieties within *Stachyurus*, with seven species from China and one species plus three varieties from the Japanese Archipelago and Korean Peninsula (Yang and Stevens, 2007; Ohba, 1999; Oh et al., 2021). These plants typically grow along moist forest edges or in stream valleys, and several species are commonly used in mountain greening due to their ecological adaptability. In horticulture, *Stachyurus* is valued for its pendulous racemes that bloom in early spring. *Stachyurus praecox* from Japan and the slightly later-flowering *S. chinensis* from China are widely cultivated as ornamental plants in temperate regions due to their early flowering. Medicinally, the dried stem pith of *Stachyurus* (known as “Xiao Tong Cao” in traditional Chinese medicine) is used for diuresis, alleviating urinary disorders, and promoting lactation postpartum (Chinese Pharmacopoeia Commission, 2020). Recent studies have found that the extracts of *S. himalaicus* and *S. chinensis* have anti-inflammatory properties (Shen et al., 1998), while triterpenoids isolated from *S. himalaicus* demonstrate potential to inhibit tumor cell proliferation *in vitro* (Wang et al., 2010; Shanmugam et al., 2012).

The taxonomy of the genus *Stachyurus* has long been contentious due to significant morphological variation and continuous trait variation across species (Li, 1943; Tang et al., 1983; Shan, 1999; Yang and Stevens, 2007). Phylogenetic studies of the genus revealed that traditional classification based on morphological traits—such as leaf shape and number of leaf-margin teeth—has been complicated by extensive intraspecific variation in key characters, resulting in ambiguous interspecific boundaries and contested species delimitation (Wei et al., 2002; Zhu, 2006). Current molecular phylogenetic studies of *Stachyurus* are limited to partial sequence data from a few individuals, and a comprehensive genus-wide phylogeny has yet to be reconstructed (Feng et al., 2020). Genetic resources from chloroplast genomes, which reflect maternal evolutionary history, remain scarce. This knowledge gap impedes robust resolution of hybridization events and mechanisms of geographic differentiation.

Chloroplasts, originating from photosynthetic bacteria, play vital roles in plant survival, adaptation, and evolution (Zhang et al., 2020). Although significantly smaller than the nuclear genome, the chloroplast genome (cpDNA) encodes proteins directly involved in photosynthesis, carbon/nitrogen fixation, and biosynthesis of starch, pigments, fatty acids, and amino acids (Dobrogojski et al., 2020). Compared to the nuclear genome, cpDNA exhibits greater structural and compositional stability: a typical angiosperm cpDNA is a circular double-stranded molecule comprising two inverted repeats (IRs) separated by large single-copy (LSC) and small single-copy (SSC) regions, forming a conserved quadripartite structure with 110–130 genes (Sugiura, 1992; Daniell et al., 2016). Due to its low recombination rate, slow nucleotide substitution rate, and uniparental inheritance, cpDNA data are widely used to resolve plant phylogenies. Structural variations—such as IR expansion/contraction, gene/intron gains/losses, and simple sequence repeats (SSRs)—also provide critical insights into genome evolution (e.g., Sabir et al., 2014; Keller et al., 2017). Recent advances in sequencing technologies and analytical tools have dramatically improved cpDNA acquisition efficiency, enabling phylogenetic studies to transition from single-gene to whole-genome scales. For instance, phylogenomic analyses based on cpDNA have successfully reconstructed evolutionary relationships at familial and generic levels in angiosperms (e.g., Cai et al., 2015; Ruhsam et al., 2015; Luo et al., 2016; Zhang et al., 2021; Zhao et al., 2019).

Here, we present complete cpDNA sequences of 25 *Stachyurus* accessions assembled from Illumina short reads and conducted comparative genomic and phylogenomic analyses to: (1) characterize the structural architecture of *Stachyurus* cpDNAs; (2) investigate variations in repetitive sequences; (3) screen hypervariable regions for species identification and phylogenetic studies; (4) reconstruct the infrageneric phylogenetic relationships; (5) explore adaptive evolution patterns in chloroplast genes. These results elucidate *Stachyurus* chloroplast evolution and phylogeny, laying the groundwork for downstream applications including hypervariable region-based marker development for barcoding and species identification, population-level phylogeographic studies to uncover hybridization and divergence patterns, and multi-genome approaches (chloroplast + nuclear) to address paraphyly, improve infrageneric resolution, and explore adaptive gene functions.

## 2 Materials and methods

### 2.1 Taxon sampling, DNA extraction, and sequencing

**Figure 1:**
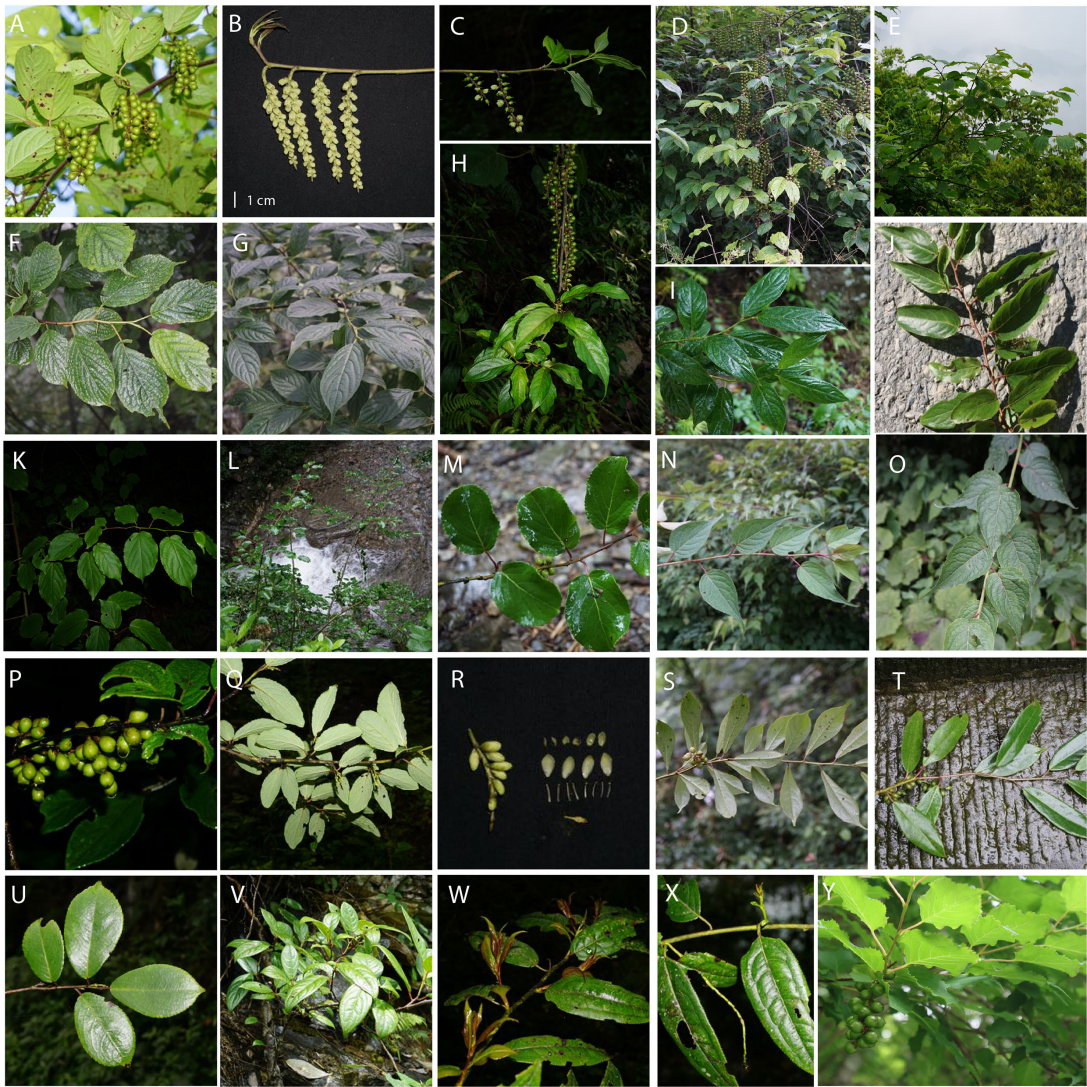
Representative photographs of *Stachyurus* accessions used in this study. **(A, B)** *S. chinensis* from Mt. Emei, Sichuan, China. **(C)** *S. chinensis* (ZG14) from Huangshan, Anhui, China. **(D)** *S. chinensis* (ZG07) from Shennongjia, Hubei, China. **(E)** *S. chinensis* (ZJY02) from Dujiangyan, Sichuan, China. **(F)** *S. chinensis* (ZJY01) from Mt. Emei, Sichuan, China. **(G)** *S. chinensis* from Jiangkou, Guizhou, China. **(H)** *S. himalaicus* (XY03) from Maguan, Yunnan, China. **(I)** *S. chinensis* (ZG08) Nanchuan, Chongqing, China. **(J)** *S. himalaicus* (XY08) from Hualian, Taiwan, China. **(K)** *S. chinensis* (ZG04) from Zhashui, Shaanxi, China. **(L, M)** *S. retusus* (AY01) from Mt. Emei, Sichuan, China. **(N, O)** *S. chinensis* (ZG04) from Baoxing, Sichuan, China. **(P, Q)** *S. retusus* (AY02) from Baoxing, Sichuan, China. **(R, S)** S. *obovatus* (DLY02) from Mt. Qingcheng, Sichuan, China. **(T)** *S. yunnanensis* (YN01) from Mt. Emei, Sichuan, China. **(U)** *S. yunnanensis* from Nanchuan, Chongqing, China. **(V)** *S. yunnanensis* (YN03) from Malipo, Yunnan, China. **(W, X)** S. *cordatulus* (DM01) from Dulongjiang, Yunnan, China. **(Y)** *S. praecox* from Ino-Cho, Japan. Figures **(J)** and **(Y)** are taken by Daiki Takahashi, and others are taken by Yu Feng.

We collected fresh leaves of *Stachyurus* from natural habitats and preserved them in silica gel desiccant. A total of 27 accessions were collected, including 25 accessions representing seven *Stachyurus* species and one variety (**Figure 1**), together with two outgroup individuals: *Euscaphis japonica* and *Turpinia montana* (**Table 1**; **Supplementary Table 1**). The identification and species assignment are according to the latest version of *Flora of China* (Yang and Stevens, 2007) and *Flora of Japan* (Ohba, 1999). Additional cpDNAs of seven species used as outgroup in phylogeny reconstruction were downloaded from NCBI (**Supplementary Table 1**). Total genomic DNA was extracted from ~110 mg silica-dried leaves using a Plant DNA kit (TIANGEN, China). Purified high-quality genomic DNA was broken into short fragments of approximately 350 bp, and paired-end (PE) libraries were constructed by adding A-tails, PCR amplification and other steps using NEBNext Ultra DNA Library Prep Kit for Illumina (New England Biolabs, Ipswich, MA, USA). Subsequent sequencing was performed via Illumina HiSeq 2500 platform by Novogene (Beijing, China), with average sequencing data of 3 Gb per sample.

**Table 1:** Sampling information and characteristics of cpDNAs for the 25 *Stachyurus* accessions used in this study.

Species	Sample code	Sample location	GenBank accession	Length (bp)				GC content (%)				Number of genes			
				LSC	SSC	IR	Total	LSC	SSC	IR	Total	PCG	tRNA	rRNA	Total
*S. retusus*	AY01	Mt. Emei, Sichuan, China	PX229812	90971	18870	26439	162719	35.1%	31.4%	42.6%	37.1%	86	37	8	131
*S. retusus*	AY02	Baoxing, Sichuan, China	PX223372	91092	18858	26433	162816	35.0%	31.5%	42.6%	37.1%	86	37	8	131
*S. chinensis*	ZJY01	Mt. Emei, Sichuan, China	PX223373	90954	18870	26438	162700	35.1%	31.4%	42.6%	37.1%	86	37	8	131
*S. chinensis*	ZJY02	Dujiangyan, Sichuan, China	PX229831	90910	18899	26445	162699	35.0%	31.4%	42.6%	37.1%	86	37	8	131
*S. obovatus*	DLY02	Mt. Qingcheng, Sichuan, China	PX223374	90941	18869	26439	162688	35.1%	31.4%	42.6%	37.1%	86	37	8	131
*S. cordatulus*	DM01	Dulongjiang, Yunnan, China	PX223375	90414	18859	26512	162297	35.1%	31.4%	42.6%	37.1%	86	37	8	131
*S. himalaicus*	XY02	Tengchong, Yunnan, China	PX229813	90843	18854	26437	162571	35.0%	31.4%	42.6%	37.1%	86	37	8	131
*S. himalaicus*	XY03	Maguan, Yunnan, China	PX229814	91016	18903	26436	162791	35.0%	31.4%	42.6%	37.1%	86	37	8	131
*S. himalaicus*	XY04	Tongmai, Xizang, China	PX229815	90916	18842	26439	162636	35.0%	31.4%	42.6%	37.0%	86	37	8	131
*S. himalaicus*	XY06	Malipo, Yunnan, China	PX229816	91130	18943	26437	162947	35.0%	31.4%	42.6%	37.0%	86	37	8	131
*S. himalaicus*	XY07	Mengzi, Yunnan, China	PX229817	90897	18884	26445	162671	35.0%	31.4%	42.6%	37.1%	86	37	8	131
*S. himalaicus*	XY08	Hualian, Taiwan, China	PX229818	90846	18745	26446	162482	35.1%	31.5%	42.6%	37.1%	86	37	8	131
*S. yunnanensis*	YN01	Mt. Emei, Sichuan, China	PX229819	90864	18878	26445	162632	35.0%	31.4%	42.6%	37.1%	86	37	8	131
*S. yunnanensis*	YN03	Suiyang, Guizhou, China	PX229820	90802	18870	26445	162562	35.1%	31.4%	42.6%	37.1%	86	37	8	131
*S. praecox*	ZC01	Nagano ken, Honshū, Japan	PX229811	90801	18784	26420	162425	35.0%	31.5%	42.6%	37.1%	86	37	8	131
*S. praecox*	ZC03	Masuda shi, Honshū, Japan	PX229821	90632	18788	26422	162264	35.0%	31.5%	42.6%	37.1%	86	37	8	131
*S. praecox* var. *lancifolius*	ZC04	Kumage gun, Kyūshū, Japan	PX229822	90783	18815	26013	161624	35.0%	31.5%	42.7%	37.1%	84	37	8	129
*S. chinensis*	ZG04	Baoxing, Sichuan, China	PX229823	91092	18858	26433	162816	35.0%	31.5%	42.6%	37.1%	86	37	8	131
*S. chinensis*	ZG05	Zhashui, Shaanxi, China	PX229824	90806	18898	26437	162578	35.0%	31.4%	42.6%	37.1%	86	37	8	131
*S. chinensis*	ZG07	Shennongjia, Hubei, China	PX229825	90753	18899	26437	162526	35.1%	31.4%	42.6%	37.1%	86	37	8	131
*S. chinensis*	ZG08	Nanchuan, Chongqing, China	PX229826	90895	18882	26445	162667	35.0%	31.4%	42.6%	37.1%	86	37	8	131
*S. chinensis*	ZG10	Nanzhao, Henan, China	PX229827	90813	18898	26437	162585	35.0%	31.4%	42.6%	37.1%	86	37	8	131
*S. chinensis*	ZG11	Lushan, Jiangxi, China	PX229828	90732	18965	26446	162589	35.1%	31.3%	42.6%	37.1%	86	37	8	131
*S. chinensis*	ZG14	Huangshan, Anhui, China	PX229829	90930	18764	26445	162584	35.0%	31.4%	42.6%	37.1%	86	37	8	131
*S. chinensis*	ZG16	Nanjiang, Sichuan, China	PX229830	90945	18882	26445	162717	35.0%	31.4%	42.6%	37.1%	86	37	8	131

### 2.2 Assembly, annotation and verification of the cpDNA

Raw sequencing data were processed using fastp v0.23.2 (Chen et al., 2018) for quality control and filtering. Standard Illumina TruSeq adapter sequences were removed and low-quality bases were trimmed using default parameters. *De novo* assembly of cpDNAs was performed with GetOrganelle v1.7.5 (Jin et al., 2020) under the parameters: -R 10 -t 20 -w 0.6 -k 21,45,65,85,105 -F embplant_pt. The assembled genomes were annotated using Plastid Genome Annotator (PGA) (Qu et al., 2019), with the annotated cpDNA of *S. chinensis* (GenBank accession MT584418.1) serving as the reference. Start/stop codons were manually corrected through homologous gene referencing in Geneious v11 (Kearse et al., 2012). Finally, annotated cpDNAs were visualized with OrganellarGenome DRAW (OGDRAW) (Lohse et al., 2013).

### 2.3 Comparative genomic, repeat sequence, and codon usage analyses

Because the cpDNAs of *Stachyurus* species exhibit strong conservation in structure and gene content, seven accessions were selected to represent the primary phylogenetic clades (as detailed in the Results) and overall species diversity for comparative genomic analyses, codon usage bias evaluation, and repeat sequence characterization. To compare IR contraction/expansion across *Stachyurus* cpDNAs, we identified and visualized the boundaries of LSC, SSC, and IR regions in seven complete genomes using CPJSdraw v1.0 (Li et al., 2023). Whole-genome comparison of seven *Stachyurus* cpDNAs was performed using mVISTA (Frazer et al., 2004; Brudno et al., 2003) in Shuffle-LAGAN mode. Relative Synonymous Codon Usage (RSCU) values were calculated using CPStools (Huang et al., 2024), where: RSCU > 1 indicates codon usage frequency higher than expected; RSCU < 1 indicates codon usage frequency lower than expected; RSCU = 1 denotes neutral codon preference.

Long repetitive sequences in the cpDNAs—including forward, palindromic, reverse, and complementary repeats—were identified using REPuter (Kurtz et al., 2001) with a minimum length threshold of 30 bp and Hamming distance of 3. SSRs were detected through the MISA network tool (Beier et al., 2017; Thiel et al., 2003). The threshold for the repeat units was set as follows: mononucleotide repeats ≥ 10; dinucleotide ≥ 6; and triple, quadruple, quintuple and sextuple nucleotide repeat units were ≥ 5. These parameters are commonly employed in cpDNA SSR mining to balance sensitivity and specificity, particularly to include prevalent A/T mononucleotide stretches in cpDNA while minimizing short, less informative repeats (e.g., as used in multiple angiosperm cpSSR studies; see examples in Zhang et al., 2023; Zhou et al., 2021).

### 2.4 Identification of molecular markers

To identify hypervariable regions potentially suitable for species identification and phylogenetic studies in *Stachyurus*, the 25 complete cpDNAs within this genus were aligned using MAFFT v7 (Katoh and Standley, 2013). Nucleotide diversity (Pi) values were calculated using DnaSP v5.10 (Librado and Rozas, 2009) with a sliding window size of 600 bp and step size of 200 bp, following parameters commonly applied in cpDNA analyses (e.g., Feng et al., 2022). Windows with Pi values > 0.006 were identified as hypervariable regions.

### 2.5 Phylogenetic analysis

To resolve phylogenetic relationships within *Stachyurus*, we incorporated cpDNA sequences from 25 *Stachyurus* accessions and 9 outgroup taxa (**Supplementary Table 1**). Phylogenetic reconstruction was performed using maximum likelihood (ML) analysis based on complete cpDNAs and coding sequences. For the former dataset, MAFFT v7 was used to obtain the alignment of 34 complete cpDNAs. For the latter, coding sequences for 78 genes shared by these 34 cpDNAs were extracted and aligned using MAFFT v7, and then concatenated by FASconCAT-G v1.05 (https://github.com/PatrickKueck/FASconCAT-G). ML phylogenetic trees were subsequently reconstructed using IQ-TREE v2.0.3 (Bui et al., 2020) with 1,000 Ultrafast Bootstrap (UFBoot; -bb 1000) replicates and 1000 Shimodaira-Hasegawa approximate Likelihood Ratio Tests (SH-aLRT; -alrt 1000). Pairwise neighbor-joining (NJ) genetic distances were calculated to quantify sequence differentiation among individuals using the complete cpDNAs and the concatenated protein-coding sequences of 25 *Stachyurus* accessions in Geneious v11.

### 2.6 Selective pressure analysis

To investigate selective pressures on protein-coding genes (PCGs) in *Stachyurus*, we estimated nonsynonymous (*d*_N_) and synonymous (*d*_S_) substitution rates using the CODEML program implemented in PAML v4.9 (Yang, 2007). The branch-site model was applied (model = 2, NSsites = 2), with CodonFreq = 2 (F61 empirical codon frequencies), runmode = 0 (user-provided tree), seqtype = 1 (codons), icode = 0 (universal genetic code), and other default settings. The *d*_N_/*d*_S_ ratio (*ω*) typically equals 1 under neutral evolution, with *ω* > 1 indicating positive selection and *ω* < 1 suggesting purifying selection. The phylogenetic tree including all 34 species was used as the input topology for CODEML. Codon-aligned nucleotide sequences, used as input sequences for CODEML, were generated by PAL2NAL (Suyama et al., 2006) under peptide alignment guidance. To determine whether each shared PCG experienced different evolutionary forces across lineages, we ran branch-site models: a one-ratio model (null hypothesis; *ω*_0_) where all branches share the same *ω*, and a two-ratio model (alternative hypothesis) where foreground branches (*Stachyurus* spp.; *ω*_f_) have distinct ω compared to background branches (*ω*_b_). Likelihood ratio tests using χ² distribution determined whether the alternative hypothesis significantly differed from the null hypothesis (Chi-square test, *p* < 0.05).

## 3 Results

### 3.1 Features of the cpDNAs of *Stachyurus*

In this study, we assembled complete cpDNAs of 25 *Stachyurus* accessions (**Table 1**). The lengths of these complete cpDNAs ranged from 161,624 bp (ZC04) to 162,947 bp (XY06), displaying the typical quadripartite structure comprising: IR regions: 26,013–26,512 bp; LSC regions: 90,414–91,130 bp; SSC regions: 18,745–18,965 bp. The GC content of *Stachyurus* cpDNAs was similar (37.0%–37.1%). The IR regions showed the highest GC content (42.6%–43.6%), followed by LSC (35.0%–35.1%) and SSC regions (31.3%–31.5%). Gene content was conserved across most accessions, with most genomes encoding 131 genes: 86 PCGs, 37 tRNA genes, and 8 rRNA genes (**Table 1**). The only exception was that *S. praecox* var. *lancifolius* (ZC04) contained only 129 genes due to loss of two copies of the *ycf15* gene in the IR regions.

### 3.2 Comparative chloroplast genomic analysis of *Stachyurus* species

**Figure 2:**
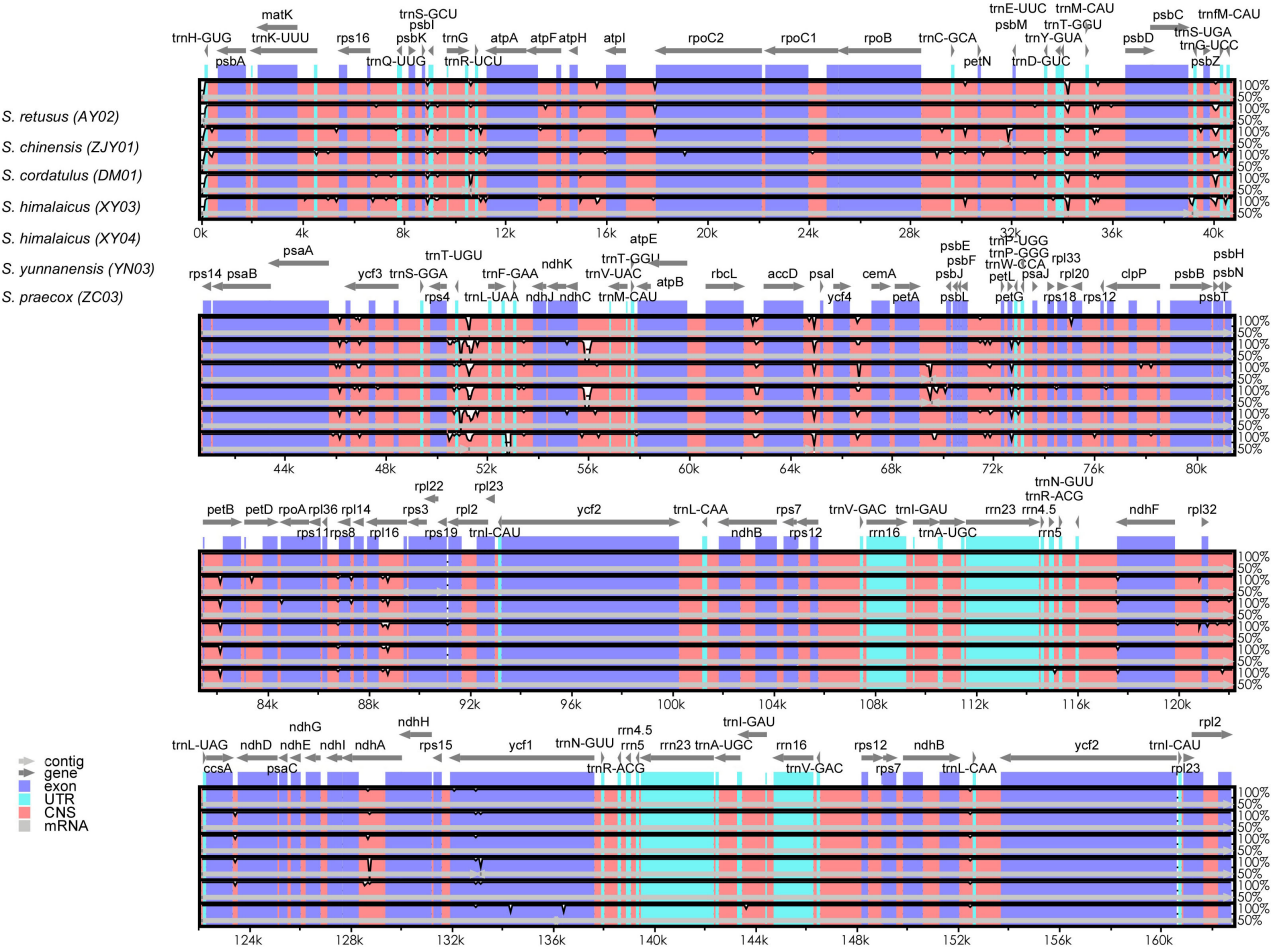
Comparative alignment of cpDNAs from seven representative *Stachyurus* accessions visualized using mVISTA. The x-axis represents the coordinate of the reference cpDNA; the y-axis lists the seven accessions. Sequence similarity of aligned regions is displayed as horizontal bars, with percent identity ranging from 50% to 100%.

**Figure 3:**
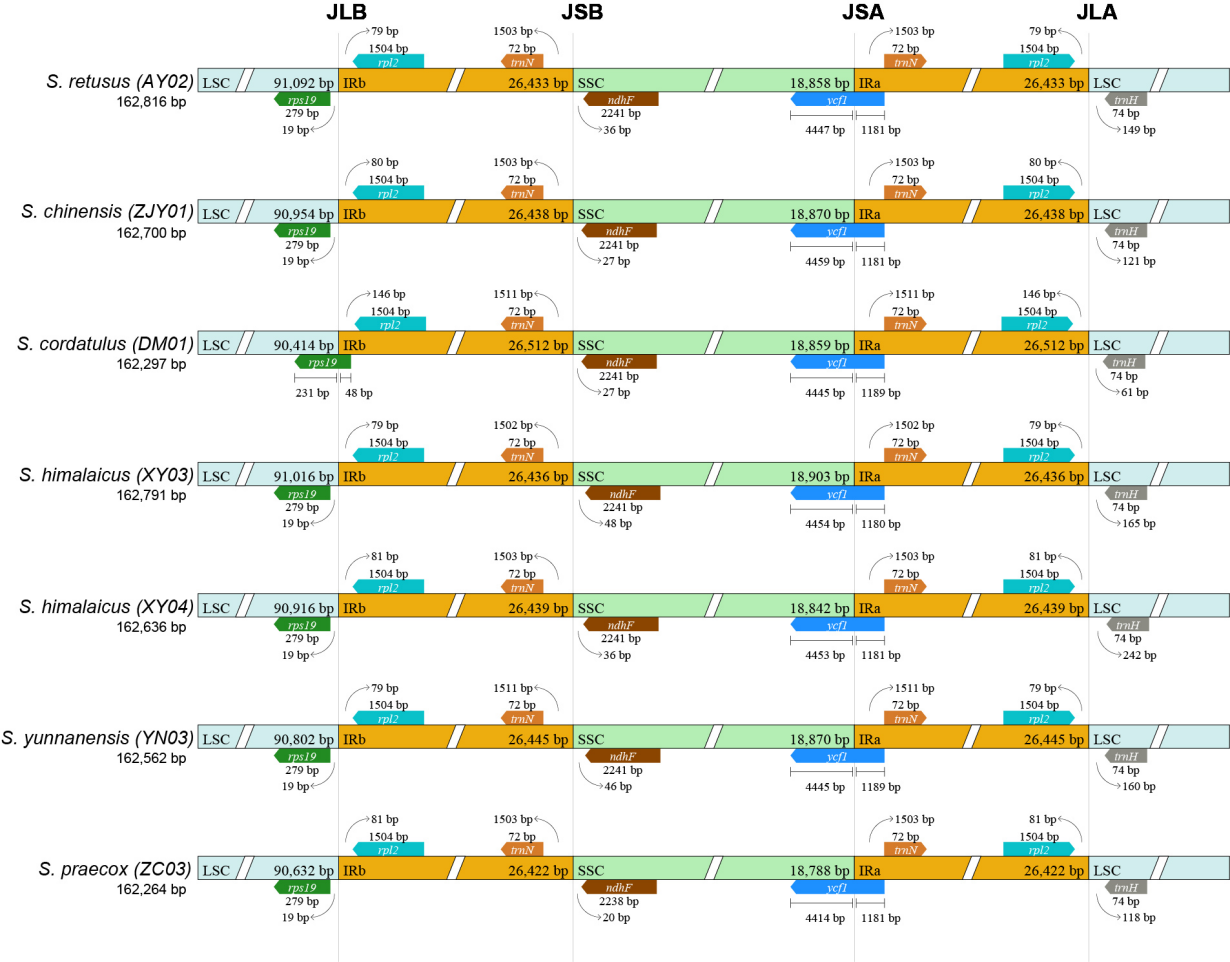
Comparison of LSC, SSC, and IR junction positions among cpDNAs of seven representative *Stachyurus* accessions. JLB denotes the LSC/IRb junction, JSB denotes the SSC/IRb junction, JSA denotes the SSC/IRa junction, and JLA denotes the LSC/IRa junction.

Comparative genomic analyses revealed high sequence conservation among the seven *Stachyurus* species. Evaluations using mVISTA highlighted stronger conservation in coding regions and intronic regions compared to non-coding regions, where substantial sequence divergence was observed (**Figure 2**). A comparative analysis of the IR/SC boundary regions in seven cpDNAs of *Stachyurus* species revealed subtle variations in the lengths of genes flanking the IR/SC junctions and the distances between these junctions and adjacent genes (**Figure 3**). The adjacent genes *rps19* and *rpl2* flank the LSC/IRb (JLB) boundary and are highly conserved across the examined species. However, in *S. cordatulus* (DM01), the *rps19* gene spans the boundary, extending 48 bp into the IRb region. The flanking genes *trnN* and *ndhF* were positioned conservatively on either side of the IRb/SSC (JSB) boundary. The gene *ycf1* spanned the SSC/IRa (JSA) boundary in all species, with approximately one-third of its sequence located in the IRa region. The length of *ycf1* in the SSC region ranged from 4,414 to 4,459 bp, while the IRa portion varied between 1,180 and 1,189 bp. Similarly, the genes *rpl2* and *trnH* were conservatively positioned on either side of the IRa/LSC (JLA) boundary.

### 3.3 Characteristics of repetitive sequences

**Figure 4:**
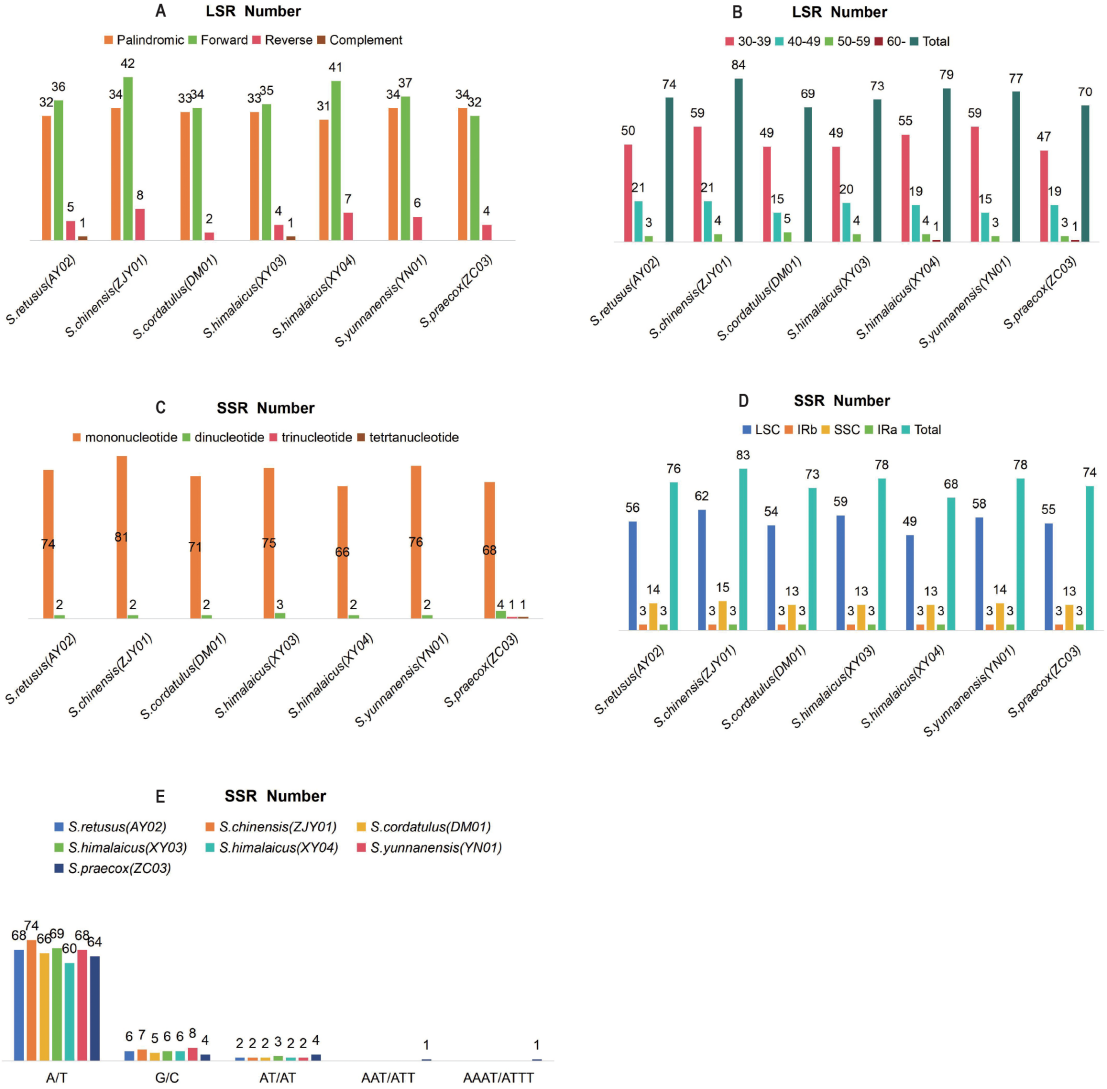
Patterns of long sequence repeats (LSRs) and simple sequence repeats (SSRs) in seven representative *Stachyurus* accessions. **(A)** Type and abundance of LSRs. **(B)** Length distribution of LSRs. **(C)** Number and abundance of SSR motifs. **(D)** Regional distribution of SSRs in the LSC, SSC, and IR regions. **(E)** Types and abundance of SSR motifs.

We identified 526 dispersed repeats (excluding IR-associated repeats) across the cpDNAs of seven *Stachyurus* accessions using REPuter (**Figures 4A, B**; **Supplementary Table 2**). The abundance and size distributions of these repeats varied significantly: forward repeats were the most frequent, followed by palindromic and reverse repeats, while complement repeats were extremely rare (only one detected in each of *S. retusus* (AY02) and *S. himalaicus* (XY03); **Figure 4A**). The total number of dispersed repeats per species ranged from 69 (*S. cordatulus* [DM01]) to 84 (*S. chinensis* [ZJY01]). Size classification revealed that 368 repeats (70%) were 30–39 bp in length, 130 (24.7%) were 40–49 bp, 26 (4.9%) were 50–59 bp, and only 2 (0.4%) exceeded 60 bp, demonstrating a strong bias toward shorter repeat motifs (**Figure 4B**). A total of 530 SSR loci spanning four types were identified, with counts ranging from 68 (*S. himalaicus* [XY04]) to 83 (*S. chinensis* [ZJY01]) per accession (**Figure 4C**). All species exhibited mono- and di-nucleotide repeats, while tri- and tetra-nucleotide repeats were uniquely detected in *S. praecox* (ZC03). Spatial distribution analysis revealed 74% of SSRs localized in the LSC region, 18% in the SSC region, and 8% in the IR regions (**Figure 4D**). Among mono-nucleotide SSRs, A/T repeats predominated, followed by G/C repeats. The most frequent di-nucleotide repeats were AT/AT, while a single tri-nucleotide (AAT/ATT) and tetra-nucleotide (AAAT/ATTT) repeat were each detected only once (**Figure 4E**).

### 3.4 Codon usage analysis

**Figure 5:**
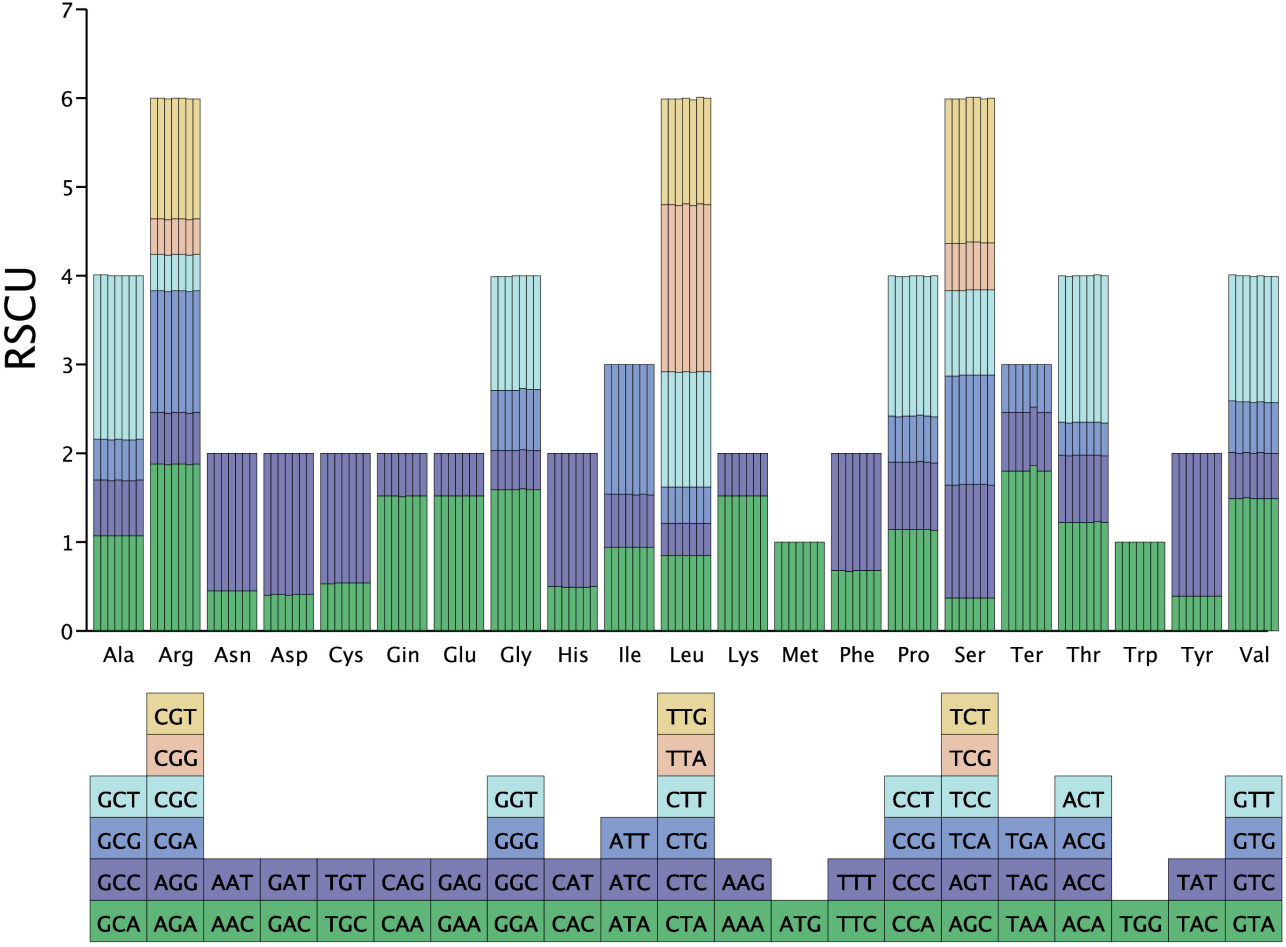
Relative synonymous codon usage (RSCU) values for protein-coding genes in seven representative *Stachyurus*. The x-axis shows amino acids and their corresponding synonymous codons; the y-axis indicates RSCU values. Histogram bars are colored according to the codon. From left to right for each amino acid: *S. retusus* (AY02), *S. chinensis* (ZJY01), S*. cordatulus* (DM01), *S. himalaicus* (XY03), *S. himalaicus* (XY04), *S. yunnanensis* (YN03), *S. praecox* (ZC03).

Codon usage analysis of cpDNAs from seven *Stachyurus* accessions was performed by concatenating 78 protein-coding regions. The results revealed highly consistent codon usage preferences across species, with minimal interspecific variation. A total of 64 codons, including three stop codons (UAA, UAG, UGA), were identified, encoding all PCGs (**Figure 5**). Of these, 50% terminated with A/T nucleotides and 50% with G/C. RSCU analysis showed that all amino acids, except methionine (Met) and tryptophan (Trp) (each encoded by a single codon with RSCU = 1), were represented by 1 to 6 synonymous codons. Notably, 30 codons had RSCU values >1, indicating preferred usage, while 32 codons had RSCU values <1, indicating underrepresentation (**Figure 5**). These findings suggest strong evolutionary conservation and a balanced codon usage pattern with no pronounced bias in the cpDNAs of *Stachyurus* species.

### 3.5 Identification of candidate molecular markers

**Figure 6:**
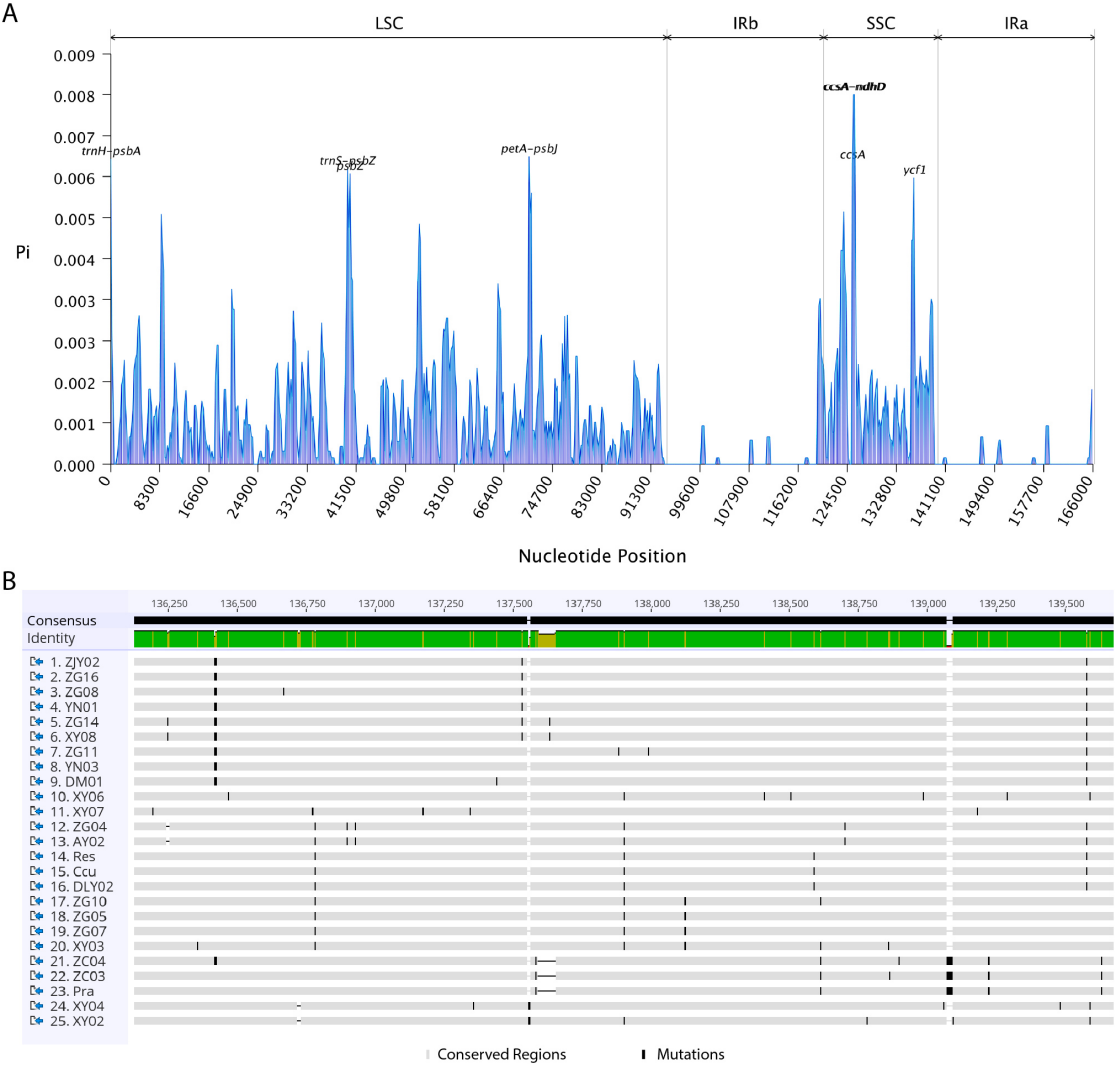
Nucleotide polymorphism patterns in the cpDNAs of 25 *Stachyurus* accessions. **(A)** Sliding-window analysis of nucleotide diversity (or polymorphism) across the aligned cpDNAs. Window size: 600 bp; step size: 200 bp. The x-axis represents the position of the window midpoint in the alignment; the y-axis shows nucleotide diversity (Pi) values. Hypervariable regions are labeled above the major peaks. **(B)** Sequence alignment visualization of a representative hypervariable region (*ycf1*). Gray blocks indicate conserved (invariable) regions; black blocks highlight variable sites (mutations/SNPs).

Sliding window analysis of all 25 *Stachyurus* cpDNAs across 600-bp windows spanning their entire cpDNAs revealed nucleotide diversity (Pi) values ranging from 0 to 0.00776 (mean = 0.00087; **Figure 6A**). Lower polymorphism was observed in IR regions compared to LSC and SSC regions, reinforcing the evolutionary conservation of IR regions. Notably, seven hypervariable loci (Pi > 0.006; **Figure 6B**, **Supplementary Table 4**) were identified predominantly in intergenic regions, with four (*trnH*-*psbA*, *trnS*-*psbZ*, *psbZ*, and *petA*-*psbJ*) localized in the LSC and three (*ccsA*-*ndhD*, *ccsA*, and *ycf1*) in the SSC. These mutation hotspots exhibit exceptional sequence divergence, positioning them as robust molecular markers for species identification and population genetic studies in *Stachyurus*.

### 3.6 Phylogenetic relationships

**Figure 7:**
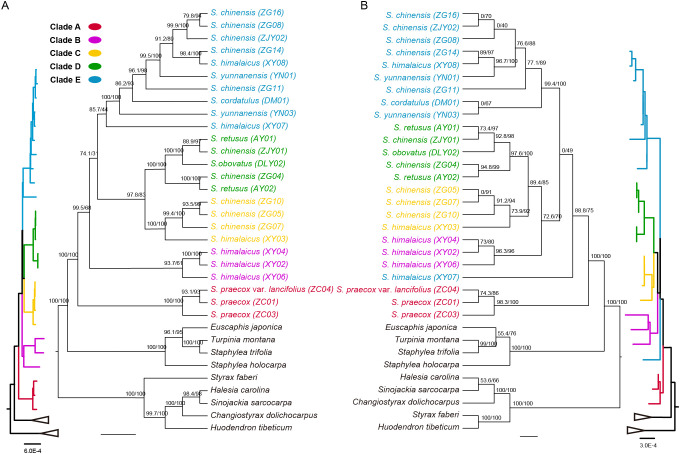
Maximum-likelihood phylogenetic trees of 25 *Stachyurus* accessions together with nine outgroup taxa, reconstructed from complete cpDNAs and concatenated protein-coding sequences. **(A)** Phylogeny inferred from the complete cpDNAs. **(B)** Phylogeny inferred from the concatenated alignment of shared protein-coding genes. Branch support was assessed using ultrafast bootstrap approximation (UFBoot) and Shimodaira–Hasegawa approximate likelihood ratio test (SH-aLRT); values are indicated adjacent to nodes. Major clades are distinguished by colored branch segments and tip labels.

The phylogenetic trees, inferred using the maximum likelihood (ML) method based on complete cpDNAs and protein-coding sequences, strongly supported the monophyly of *Stachyurus* (UFBoot = 100%; SH-aLRT = 100%; **Figure 7**). Individuals from Staphyleaceae formed a sister clade to *Stachyurus* (UFBoot = 100%; SH-aLRT = 100%). The phylogenetic tree based on complete cpDNAs divided all *Stachyurus* species into five distinct clades (**Figure 7A**). *Stachyurus praecox* and *S. praecox* var. *lancifolius* from the Japanese Archipelago formed a clade (Clade A; UFBoot = 100%; SH-aLRT = 100%) sister to a super clade containing all other individuals collected from mainland China and Taiwan Island (UFBoot = 100%; SH-aLRT = 100%). However, the relationships within this super clade were unstable and supported by relatively lower values. Clade B (UFBoot = 93.7%; SH-aLRT = 61%), which includes three *S. himalaicus* individuals (XY02, XY04, and XY06), was weakly supported as sister to Clade C+D+E (UFBoot = 74.1%; SH-aLRT = 31%). Clade C (UFBoot = 100%; SH-aLRT = 100%) and Clade D (UFBoot = 100%; SH-aLRT = 100%) formed a clade (UFBoot = 97.8%; SH-aLRT = 83%) that is sister to Clade E. Clade C comprises individuals from two widely distributed species (*S. himalaicus* and *S. chinensis*), while Clades D and E included both widely distributed and narrowly endemic species (such as *S. retusus* and *S. obovatus*). Notably, several widely distributed species, including *S. himalaicus*, *S. chinensis*, *S. yunnanensis*, and *S. retusus*, were found to be paraphyletic. Accessions of the same species were scattered across multiple clades, with several nodes receiving moderate to strong support (e.g., UFBoot ≥ 93.7% for Clade B; UFBoot = 100% for Clades C and D). This paraphyly is further supported by near-zero genetic distances between certain accessions belonging to different species (e.g., ZJY01 (*S. chinensis*) and DLY02 (*S. obovatus*); **Supplementary Table 5**).

The topology based on two datasets (complete cpDNAs and protein-coding sequences) revealed slight differences in support values and relationships. While most nodes in the phylogenetic tree based on complete cpDNAs received support values exceeding 90%, that based on protein-coding sequences exhibited lower support (**Figure 7B**). A notable discordance between the datasets involves the placement of *S. himalaicus* (XY07), which appeared as sister to a clade containing all remaining individuals from China (UFBoot = 88.8%; SH-aLRT = 75%) in the protein-coding tree, rather than being part of Clade E as in the complete cpDNA tree. Minor discordances also occurred within Clade E, particularly in the positions of *S. cordatulus* (DM01), *S. yunnanensis* (YN03), and *S. chinensis* var. *cuspidatus* (ZJY02). Notably, both phylogenetic trees revealed several accessions with near-zero-length terminal branches (**Figure 7**), indicating very few detectable sequence variations relative to their sister clades. Pairwise NJ distance analysis of the complete cpDNAs identified five pairs with zero genetic distance (**Supplementary Table 5**). For example, accessions AY01, ZJY01, and DLY02 shared nearly identical cpDNAs. In contrast, the concatenated protein-coding sequences exhibited even more zero-distance pairs (**Supplementary Table 6**), suggesting lower sequence variation in the coding regions compared to the complete cpDNAs.

### 3.7 Selection pressure analysis

**Figure 8:**
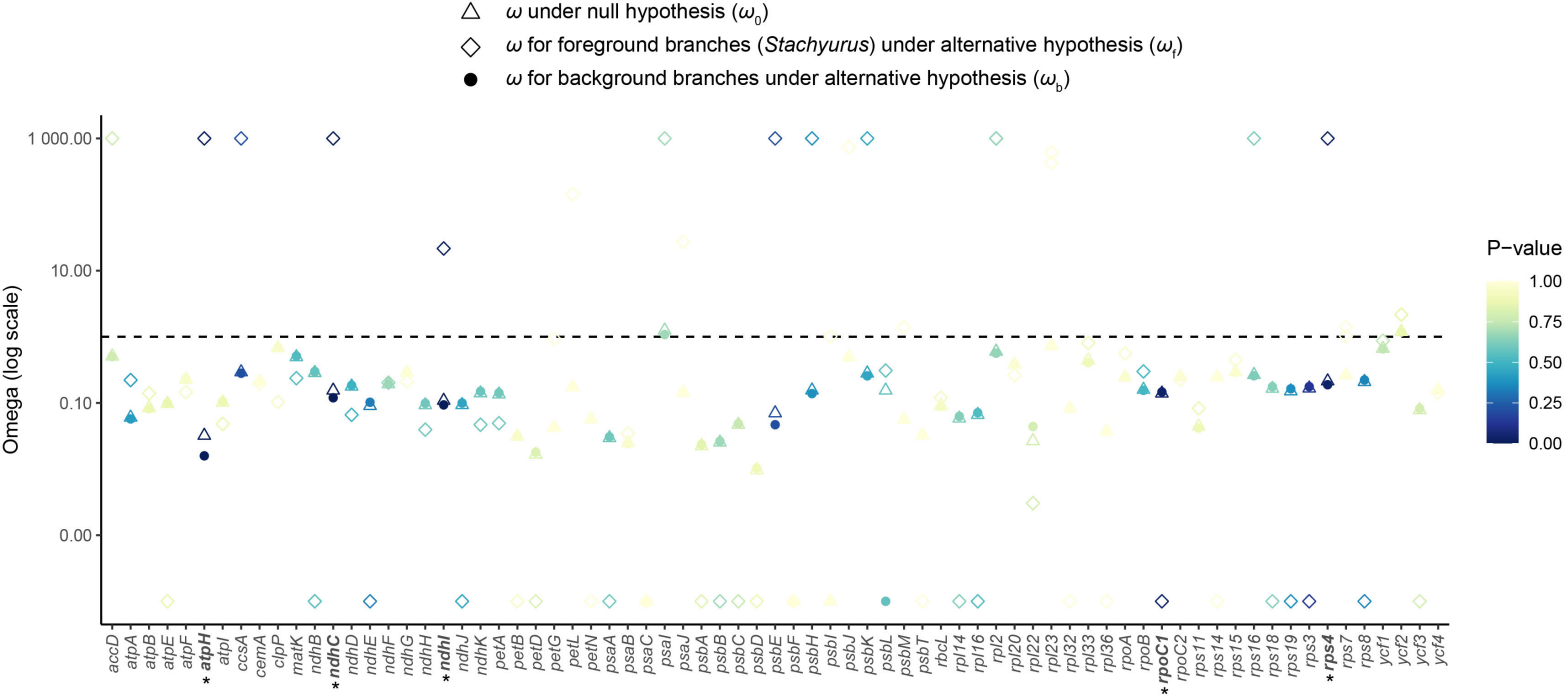
Ratios of nonsynonymous to synonymous substitutions (*d*_N_/*d*_S_; *ω*) for protein-coding genes in *Stachyurus*. Gene names in bold with an asterisk (*) indicate statistically significant evidence of selection (*P* < 0.05) based on likelihood ratio tests between the null and alternative models.

We conducted selection pressure analysis on 78 PCGs (**Figure 8**; **Supplementary Table 3**). Most genes were under purifying selection (*ω* < 1). Using likelihood ratio tests, we identified five genes exhibiting significantly distinct selection pressures within *Stachyurus* (*p* < 0.05). Among these, four genes (*rps4*, *ndhC*, *atpH*, and *ndhI*) showed strong positive selection signal (*ω*_f_ > 1), and demonstrated accelerated evolutionary rates in *Stachyurus* compared to background branches (*ω*_f_ > *ω*_b_, *p* < 0.05). One gene (*rpoC1*) showed stronger negative selection signal (*ω*_f_ < *ω*_b_, *p* < 0.05) in *Stachyurus* than background branches.

## 4 Discussion

### 4.1 Variation and evolution of the cpDNAs in *Stachyurus*

The cpDNAs of *Stachyurus* species exhibit a typical quadripartite structure with no large-scale structural variations detected. The genome sizes of these species are highly similar, ranging from 161,624 bp (ZC04) to 162,947 bp (XY06). Other genomic features—including the lengths of LSC, SSC, and IR regions, expansion/contraction of IR boundaries, gene content, GC composition, and patterns of codon usage—also display highly conserved characteristics within this genus. These results are similar to previously reported cpDNAs of *Stachyurus* species (Su et al., 2020; Chen et al., 2025). Despite the conserved characteristics described above, it is noteworthy that previous studies have demonstrated that expansion/contraction of IR regions significantly contributes to cpDNA size variation (Zhu et al., 2016; Abdullah et al., 2020). In *S. cordatulus* (DM01), the *rps19* gene spans the JLB (LSC/IRb) boundary, resulting in a slight expansion of the IR and a corresponding contraction of the LSC by 67 bp (**Figure 3**). Although IR expansion typically increases overall genome size, DM01 has a relatively short total cpDNA length (162,297 bp vs. average 162,583 bp) and the shortest LSC region (90,414 bp vs. average 90,817 bp; **Table 1**). This indicates that the modest IR expansion was more than offset by other structural changes, most likely sequence deletions in the LSC regions. Additional factors contributing to size variation include gene loss and repeat content. For example, *S. praecox* var. *lancifolius* (ZC04) has lost two copies of *ycf15* gene in the IR regions, leading to a significant reduction in cpDNA length (161,624 vs. 162,583 average; **Table 1**). Such gene losses are known to cause measurable contractions in cpDNAs. Regarding repeat sequence analysis, variations in SSR types and numbers do not substantially contribute to length differences among the sampled genomes (**Figure 4**). In contrast, LSRs appear to play a more noticeable role. *S. chinensis* (ZJY01) and *S. himalaicus* (XY04) possess the highest numbers of LSRs, correlating with their relatively longer cpDNAs (**Figure 4**, **Table 1**).

Positive selection plays a pivotal role in driving molecular adaptation and functional divergence of plastid genes under environmental stress, whereas purifying selection (negative selection) acts as a pervasive evolutionary force maintaining sequence conservation across extended evolutionary timescales (Ometto et al., 2012; Zheng et al., 2025). In this study, the majority of genes exhibited *ω* (*d*_N_/*d*_S_ ratio) values < 1 across both focal species and their background branches (**Figure 8**), which is similar to previously reported patterns (Gu et al., 2023; Feng et al., 2022). Four genes under significant positive selection were identified in *Stachyurus* (**Figure 8**), including those involved in genetic systems (*rps4*) and photosynthesis (*ndhC*, *atpH*, *ndhI*). Their adaptive variations likely correlate with ecological adaptations of *Stachyurus* species to heterogeneous light intensities and elevational gradients (e.g., Chen et al., 2022; Ma et al., 2024).

### 4.2 Implications for phylogenetic relationships and taxonomy within *Stachyurus*

Stachyuraceae is an East Asian-endemic family of small shrubs or trees that comprises the single genus *Stachyurus*. The family diverged from its sister group (Crossosomataceae or Staphyleaceae) during the Paleocene to Eocene (Zhu et al., 2006; Feng et al., 2020), while most of its extant species diversity arose during the Late Miocene (Feng et al., 2020). Our phylogenetic trees, consistent with previous studies, strongly support the monophyly of *S. praecox* and its varieties from Japan (**Figure 7**; Su et al., 2020; Feng et al., 2020). In contrast, evolutionary relationships among Chinese *Stachyurus* species are far more complex, exhibiting widespread paraphyly. The majority of species diversity within the genus is concentrated in southwest China, a global biodiversity hotspot. The recent uplift of the Qinghai–Tibet Plateau and adjacent regions, together with shifts in monsoon patterns, has promoted rapid radiations and facilitated frequent hybridization and incomplete lineage sorting in many plant genera, including *Rhododendron* (Yan et al., 2015), *Primula* (He et al., 2019), *Campylotropis* (Feng et al., 2022), and *Pedicularis* (Yu et al., 2015). These same processes likely explain the extensive paraphyly observed within the Chinese *Stachyurus* clade.

Historically, *Stachyurus* was classified into two sections based on leaf habit: sect. *Callosurus* (evergreen) and sect. *Gymnosurus* (deciduous) (Franchet, 1898). Feng et al. (2020) recovered distinct clades corresponding to evergreen and deciduous species in their nuclear phylogeny. However, our cpDNA-based phylogeny fails to support the monophyly of either group (**Figure 7**), revealing substantial incongruence between plastid and nuclear topologies. In addition, while Feng et al. (2020) found that most narrowly endemic species are nested within widely distributed taxa, our analyses recovered extensive paraphyly not only in widespread species such as *S. praecox*, *S. yunnanensis*, *S. himalaicus*, and *S. chinensis*, but also in the narrowly endemic *S. retusus* (**Figure 7**). These topological discrepancies strongly suggest widespread chloroplast capture events driven by interspecific hybridization. A clear signature of chloroplast capture is that geographically proximate individuals from morphologically distinct species frequently cluster together in the chloroplast phylogeny. For example, the deciduous species *S. retusus* (AY01) and *S. chinensis* (ZJY01), both collected from Mt. Emei in Sichuan, China, share nearly identical cpDNAs (near-zero NJ distance; **Supplementary Table 5**), despite their clear morphological differences. This clade is sister to the evergreen species *S. obovatus* (DLY02), collected from Mt. Qingcheng (approximately 120 km away), a relationship that contrasts sharply with the nuclear phylogeny, in which *S. obovatus* clusters with other evergreen species (Feng et al., 2020). Additionally, our chloroplast phylogeny recovered a strongly supported sister relationship between AY02 (*S. retusus*; **Figures 1P, Q**) and ZG04 (*S. chinensis*; **Figures 1N, O**), both collected from the same valley in Baoxing County, Sichuan, China (**Figure 7**), despite their markedly different morphologies. This topology is congruent with the nuclear phylogeny reported by Feng et al. (2020). However, according to the same study, these two accessions (previously identified as *S. szechuanensis* and *S. chinensis* var. *latus* by Shan, 1999) were inferred to be the hybridization progenitors of *S. retusus* (AY01, Mt. Emei) and *S. chinensis* (ZG05, Zhashui, Shaanxi). The close sister relationship observed here thus represents another clear case of chloroplast capture resulting from hybridization.

Another notable finding is the topological incongruence between the phylogenies reconstructed from the complete cpDNAs and the concatenated protein-coding sequences. Overall, sequence divergence among *Stachyurus* cpDNAs is extremely low (pairwise NJ distance ≤ 0.019, mean = 0.0011; **Supplementary Table 5**), and even lower among the coding sequences (pairwise NJ distance ≤ 0.016, mean = 0.0009; **Supplementary Table 6**). A representative example of this conflict is the placement of *S. himalaicus* (XY07). In the complete cpDNA phylogeny, this accession belongs to Clade E, whereas in the coding-sequence tree, the sister relationship between Clade E and Clades B + C + D receives zero bootstrap support and the branch leading to their common ancestor is nearly zero in length (**Figure 7B**). These patterns suggest that the three lineages diverged nearly simultaneously, resulting in insufficient phylogenetic signal to confidently resolve their branching order. A similar lack of resolution was observed for several other relationships, most likely due to rapid radiation and limited phylogenetic signal in the protein-coding regions.

These patterns raise important questions regarding species boundaries within *Stachyurus*. Do most species in the genus represent incipient species characterized by very low interspecific divergence and largely transparent species boundaries? Are the narrowly endemic taxa distinct evolutionary lineages, or merely regional morphological variants of more widely distributed species? Resolving these uncertainties will require future integrative studies that combine dense population-level sampling with multidimensional datasets, including nuclear genomic, morphological, and ecological data.

## 5 Conclusions

In this study, we sequenced and assembled the complete cpDNAs of 25 *Stachyurus* accessions, representing seven species and one variety. Comparative analyses revealed a high degree of conservation in genome size, structure, IR boundary dynamics, gene content, GC content, and repeat patterns. Nevertheless, subtle variations driven by IR boundary shifts, gene loss (particularly *ycf15*), and differences in long sequence repeats collectively account for the observed cpDNA length variation. We also identified seven hypervariable regions as promising molecular markers for future phylogenetic and population genetic studies. Notably, four genes (*rps4*, *ndhC*, *atpH*, and *ndhI*) exhibited signatures of positive selection and rapid evolution, offering potential insights into adaptive mechanisms in the genus. Phylogenetic analyses based on complete chloroplast genomes uncovered widespread paraphyly and substantial incongruence with previous nuclear dataset-based phylogenies, providing strong evidence of chloroplast capture events associated with hybridization. These results highlight the limitations of relying solely on plastid data and emphasize the importance of future integrative studies that combine nuclear genomic, morphological, and ecological data to clarify species boundaries and taxonomic relationships within *Stachyurus*.

## Data Availability

The datasets presented in this study can be found in online repositories. The names of the repository/repositories and accession number(s) can be found in the article/**Supplementary Material**.
